# Changes of Fixed Anatomical Spinopelvic Parameter in Patients with Lumbosacral Transitional Vertebrae: A Matched Pair Analysis

**DOI:** 10.3390/diagnostics11010059

**Published:** 2021-01-02

**Authors:** Henryk Haffer, Luis Becker, Michael Putzier, Mats Wiethölter, Katharina Ziegeler, Torsten Diekhoff, Matthias Pumberger, Sebastian Hardt

**Affiliations:** 1 Center for Musculoskeletal Surgery, Charité-University Medicine, 10117 Berlin, Germany; luis-alexander.becker@charite.de (L.B.); michael.putzier@charite.de (M.P.); mats-jonas.wiethoelter@charite.de (M.W.); matthias.pumberger@charite.de (M.P.); sebastian.hardt@charite.de (S.H.); 2 Clinic of Radiology, Charité-University Medicine, 10117 Berlin, Germany; katharina.ziegeler@charite.de (K.Z.); torsten.diekhoff@charite.de (T.D.)

**Keywords:** pelvic incidence, sacral table angle, pelvic radius, lumbarization, sacralization, LSTV, spinal alignment

## Abstract

Functional spinopelvic parameters are crucial for describing spinal alignment (SA), but this is susceptible to variation. Anatomically fixed pelvic shape is defined by the parameters pelvic radius (PR), pelvic incidence (PI), and sacral table angle (STA). In patients with lumbosacral transitional vertebrae (LSTV), the spinopelvic alignment may be altered by changes of these parameters and influences of SA. There have been no reports studying the relation between LSTV, four (4 LV) and six (6 LV) lumbar vertebrae, and fixed anatomical spinopelvic parameters. A retrospective analysis of 819 abdomen–pelvis CT scans was performed, identifying 53 patients with LSTV. In a matched-pair analysis, we analyzed the influence of LSTV and the subgroups 4 LV (*n* = 9) and 6 LV (*n* = 11) on PR, PI, and STA. LSTV were classified according to Castellvi classification. In patients with 6 LV, measurement points at the superior endplates of S1 and S2 were compared. The prevalence of LSTV was 6.5% (53/819), 6 LV was 1.3% (11/819), and 4 LV was 1.1% (9/819) in our study population. PI significantly increased (*p* < 0.001), STA significantly decreased (*p* < 0.001), and PR (*p* = 0.051) did not differ significantly in the LSTV group (*n* = 53). Similar findings were observed in the 4 LV subgroup, with an increase in PI (*p* < 0.021), decrease in STA (*p* < 0.011), and no significant difference in PR (*p* < 0.678). The same results were obtained in the 6 LV subgroup at measuring point S2 (true S1) PI (*p* = 0.010), STA (*p* = 0.004), and PR (*p* = 0.859), but not at measuring point S1 (true L6). Patients with LSTV, 4 LV, and 6 LV showed significant differences in PI and STA compared to the matched control group. PR showed no significant differences. The altered spinopelvic anatomy in LSTV patients need to be reflected in preoperative planning rebalancing the sagittal SA.

## 1. Introduction

Lumbosacral transitional vertebrae (LSTV) is a common congenital spinal abnormality with a widespread reported prevalence of 3.9–35.6% [1,2,3]. The spectrum of LSTV is defined by sacralization of the lowest lumbar segment or a lumbarization of the uppermost sacral segment, and was initially classified by Castellvi [4]. This radiological classification, divided into four types, includes dysplastic enlarged costal process, pseudarthrosis, osseous fusion, and a mixed type (Table 1). In general, LSTV and numerical variations of lumbar vertebrae can be over- or underestimated by incorrect diagnostics or counting [5,6,7]. The prevalence of LSTV varies from region to region, with a prevalence of 15.8% in a Chinese and 27.6% in a Central European cohort, whereas a prevalence of 35.6% was reported in an American study group [1,2,8,9]. It is reported that spinopelvic parameters are also influenced by ethnic background [10]. The LSTV prevalence is markedly influenced by the cohort, with patients who had attended a spine outpatient clinic (14.0%), patients who had undergone lumbar disc herniation surgery (16.9%), and a cohort with suspected spondyloarthritis (25.0%) showing a higher prevalence than patients in a urology outpatient department (8.1%) %) [11,12]. Incorrect identification of LSTV may lead to misdiagnosis, miscalculation of sagittal balance and spinopelvic parameters, and subsequent inappropriate surgical treatment. This clearly underlines the high relevance of this anatomical variant, and should lead to increased attention in routine clinical practice.

Spinal sagittal balance and spinopelvic alignment have become indispensable tools in the etiology, description, and treatment of spinal pathologies in recent years [13,14,15]. One of the most influential attempts to describe the sagittal profile was performed by Roussouly et al., classifying the sagittal spinal profile into four and five types, respectively, in two studies [16,17]. It has been demonstrated that patient satisfaction and adjacent segmental degeneration in lumbar fusion surgery depends on a sufficient restoration of the sagittal profile [15,18]. Schwab et al. have developed a classification based on radiological parameters and health-related quality of life (HRQOL) scores, with the aim of restoring the sagittal profile [19]. Spinopelvic parameters have not been integrated into the aforementioned Schwab classification. Therefore, they were established in the newly developed SRS–Schwab classification with the three modifiers PI-LL (pelvic incidence minus lumbar lordosis) mismatch, used in adult spinal deformity surgery planning; global alignment (sagittal vertical axis); and pelvic tilt [20]. The European Spine Study Group (ESSG) considers that the widely used SRS–Schwab classification for determining optimal sagittal alignment neglects mechanical complications. Thus, the ESSG has introduced the Global Alignment and Proportion (GAP) score, a method based on pelvic incidence for analyzing the sagittal alignment and predicting mechanical complications [21]. These comprehensive efforts highlight the substantial influence of the spinopelvic parameters, in particular constant pelvic incidence (PI), on the spinal alignment. Nevertheless, no final conclusion has yet been reached on the “optimal” formula for the reconstruction of sagittal alignment.

However, these concepts of spinopelvic alignment do not take into account both short- and long-term variations in the sagittal spinal profile [22]. Some authors therefore recommend a closer look at the anatomical aspect of the pelvis, which is considered to be the socket of the vertebral column architecture [13,14]. To adequately describe the constant pelvic shape, the combination of more than one fixed spinopelvic parameter has been discussed [23,24,25]. As the spinopelvic alignment is presumed to be variable, we therefore focus on the anatomically constant pelvic shape defining parameters pelvic radius (PR), PI, and sacral table angle (STA). It has been reported in LSTV populations that the spinopelvic parameters, such as sacral slope (SS; angle between the tangent line to the superior endplate of the sacrum and the horizontal plane), pelvic tilt (PT; angle between a line through the midpoint of the sacral plate to the femoral head axis and a vertical line), pelvic incidence (PI), and lumbar lordosis (LL) can be altered due to this anatomical variant [8,26,27]. Furthermore, there is still controversy about the change in PI in patients with six lumbar vertebrae (6 LV) [26,27]. A relevant clinical question about the standardized application of the measurement points of spinopelvic parameters in patients with numerical variants of the lumbosacral segment of the vertebral column still remains [27,28].

We compared the anatomically fixed spinopelvic parameters in the LSTV and non-LSTV cohort. An investigation on the influence of PI, PR, and STA as a function of the Castellvi classification and the clusters of four lumbar vertebrae (4 LV) and six LV (6 LV) vertebral columns was performed. We analyzed the effect of the different measuring points in patients with 6 LV. In our collective patients, we assessed the prevalence of LSTV, 4 LV, and 6 LV. The definition of normative values of the anatomically fixed spinopelvic parameters in LSTV patients can have an influence on diagnostics and therapy decisions in spinal pathologies.

## 2. Materials and Methods

A retrospective matched-pair analysis of abdomen–pelvis CT scans was performed. To determine whether the occurrence of LSTV leads to changes in the spinopelvic parameters PI, PR, and STA, we analyzed CT scans of patients with and without LSTV. The study was reported according to the guidelines of the STROBE statement.

### 2.1. Individuals

The participants were central Europeans. The study was approved by the local ethics board (ethics proposal number EA1/300/19). Included patients underwent high-resolution abdomen–pelvis CT scans with an image section from at least level L1 to the greater trochanter of the femur; these scans were acquired, due to tumor staging, detection of a bleeding source, search for an infection focus, and trauma, in our department of radiology from 2016 to 2019. Exclusion criteria were primary and metastatic malignancy of the musculoskeletal system, previous spinal or pelvic fusion surgery, pelvic fracture, known rheumatic disease, known as SIJ disease, low-dose imaging CT technique, incomplete image data. and insufficient image quality for software evaluation. 819 patients were included (mean age = 50.6 years, range = 15–92 years, with 402 female and 417 male). Fifty-three patients (mean age = 51.6 years, range 16–81 years, with 25 female and 28 male) had LSTV. These were matched with control patients from the cohort described above, using propensity score matching with a tolerance of 0.01, matching for age and gender.

### 2.2. Image Assessment

**Figure 1:**
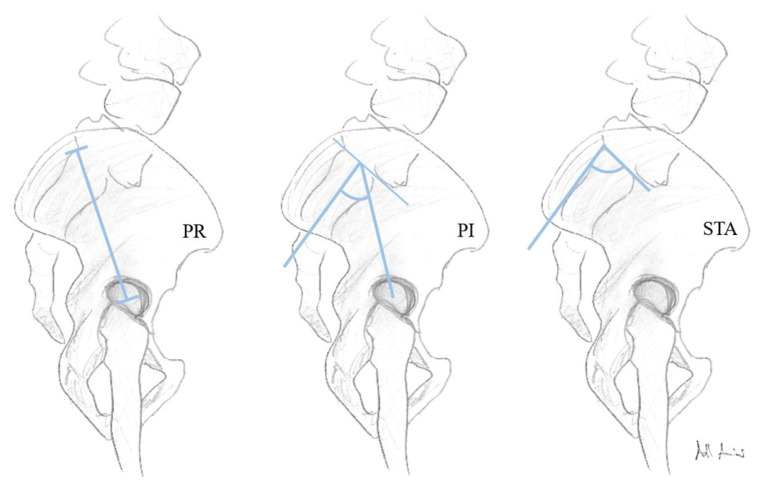
Schematic illustration of the constant anatomical parameters pelvic radius (PR), pelvic incidence (PI), and sacral table angle (STA).

**Figure 2:**
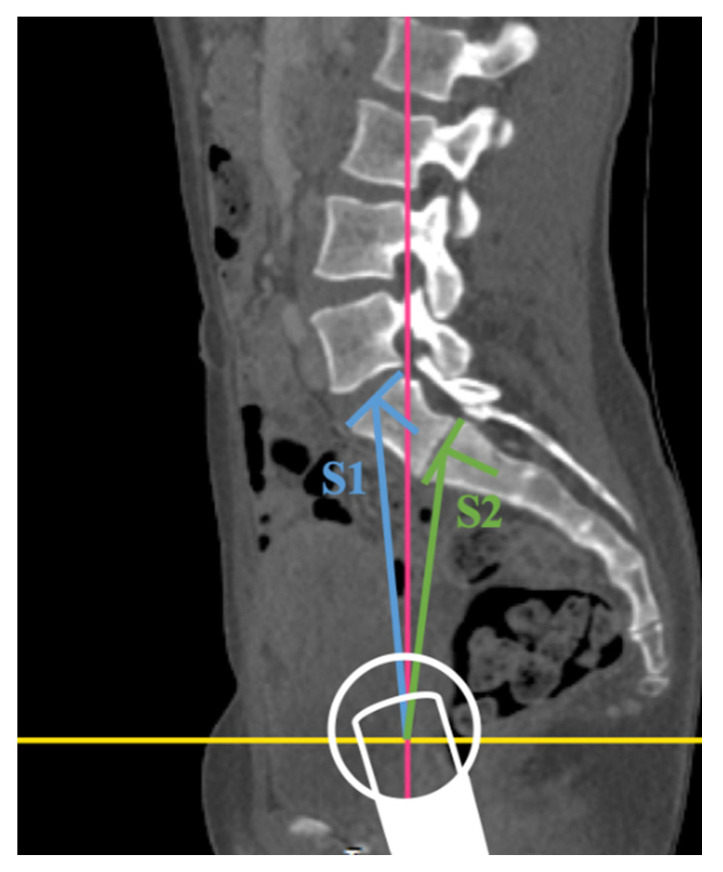
Illustration of the two measurement points S1 (true L6, blue) and S2 (true S1, green) in an LSTV patient with lumbarization and six lumbar vertebrae, classified as Catellvi type IIIa by the example of PI in the multiplanar reconstruction of an abdomen–pelvis CT (in this sectional view, the intersection of the yellow and red line represents the bicoxofemoral axis; schematic illustration of the femoral head).

**Figure 3:**
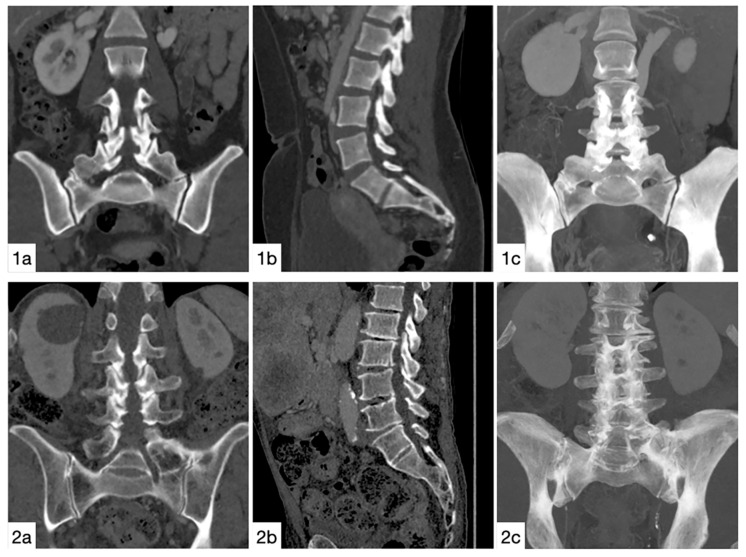
Imaging examples. Castellvi IIa: coronal (**1a**) and sagittal (**1b**) reconstructions, as well as 34 mm maximum intensity projection (**1c**). Castellvi IIIb: coronal (**2a**) and sagittal (**2b**) reconstructions, as well as 34 mm maximum intensity projection (**2c**).

**Figure 4:**
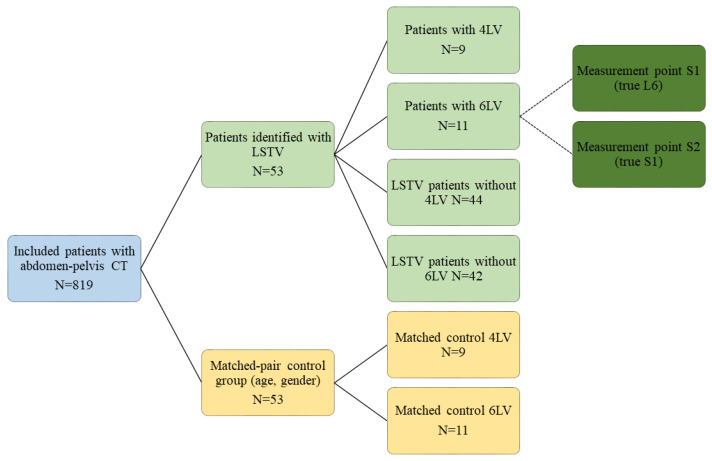
Flow chart of the patients enrolled who had lumbosacral transitional vertebrae (LSTV) (*n* = 53) and a matched-pair control group (*n* = 53), including LSTV subgroups with four (4 LV) and six (6 LV) lumbar vertebrae, LSTV subgroups without four (4 LV) or six (6 LV) lumbar vertebrae, matched-pair control groups to the four (4 LV) and six (6 LV) lumbar vertebrae groups, and the two measurement points in the 6 LV subgroup.

**Figure 5:**
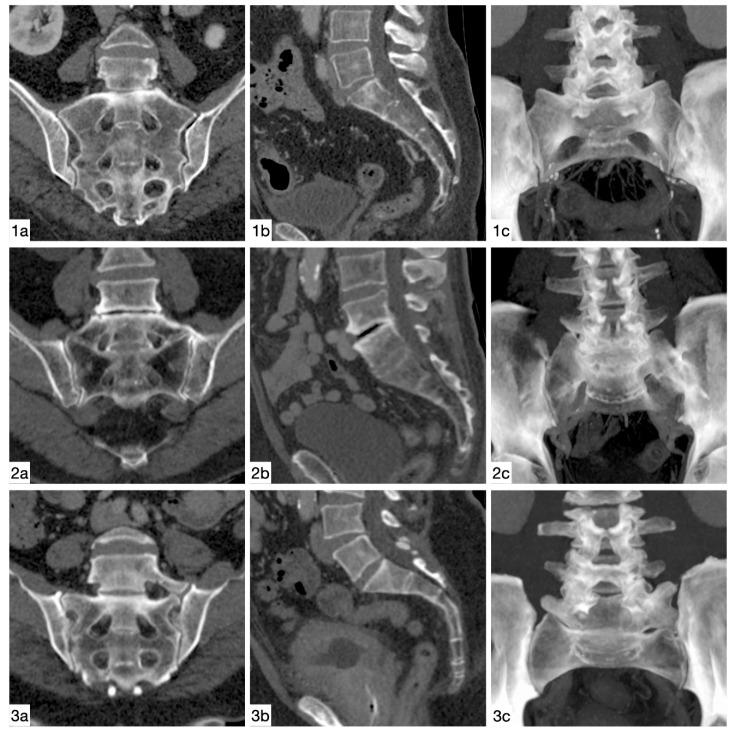
Imaging examples. Normal anatomy: oblique coronal, parallel to S2 (**1a**) and sagittal (**1b**) reconstructions, as well as 34 mm maximum intensity projection (**1c**); 4 LV: oblique coronal, parallel to S2 (**2a**), and sagittal (**2b**) reconstructions, as well as 34 mm maximum intensity projection (**2c**); 6 LV: oblique coronal, parallel to S2 (**3a**), and sagittal (**3b**) reconstructions, as well as 34 mm maximum intensity projection (**3c**).

The images were taken by an 80-row or a 320-row CT scanner (Canon Aquillon Prime and Canon Aquillon One Vision, Canon Medical Systems, Otawara, Japan) and reconstructed in an isometric volume with 1.0 mm slice thickness in a medium soft-tissue kernel with beam-hardening compensation. CT images were reconstructed with the image analysis software RadiAnt DICOM Viewer 2020.2 (Medixant, Poznan, Poland). Image analysis and measurements were independently performed by two orthopedic surgeons experienced in measuring radiological spinal parameters. The following parameters have been measured in the multiplanar reconstruction of the abdomen-pelvis CT: angle formed by a line perpendicular to the superior sacral endplate at its midpoint and a line connecting the same point to the center of the bicoxofemoral axis (PI), distance between the superior posterior angle of the superior sacral endplate and the center of the bicoxofemoral axis (PR), and the angle between the superior sacral endplate and the posterior wall of the sacrum (STA) (Figure 1). The measurements were performed in patients with six lumbar vertebrae both in S1 and S2 (Figure 2). We assumed the first vertebral body without a rib as L1 and counted it caudally. A sixth lumbar vertebra has been defined as if there is complete disc material between L6 and S1 over the entire anterior posterior diameter of the sacrum. We defined four lumbar vertebrae as osseous fusions of the fifth non-rib bearing vertebra with the sacrum. An experienced radiologist evaluated the lumbosacral transition disorders according to the Castellvi classification (Table 1 and Figure 3). We formed groups with all LSTV patients (*n* = 53) and their matched control group (*n* = 53) and subgroups with six (6 LV; *n* = 11) and four lumbar vertebrae (4 LV; *n* = 9), as well as their corresponding matched control groups and LSTV subgroups without 6 LV (*n* = 42) and without 4 LV (*n* = 44) (Figure 4 and Figure 5).

### 2.3. Statistical Analysis

All statistical analyses were performed using SPSS Version 27 (IBM Corporation, New York, NY, United States). We used the Wilcoxon signed-rank test to compare the differences between the matched groups and the Mann–Whitney U test between unrelated groups. The association between the degree of fusion (Castellvi classification) and spinopelvic parameters (PR, PI, and STA) was investigated using Kendall’s τ coefficient. Spearman’s rank correlation coefficient was used to determine the interrater reliability of the radiographic measurements. A significance level of *p* < 0.05 was assumed for all tests.

## 3. Results

### 3.1. Patients

From the 819 abdomen–pelvis CT scans included, 53 patients with LSTV were identified and matched with 53 patients by age and gender. Based on the study design, there were no significant differences in age (mean = 51.6 years, range = 16–81/86 years (LSTV/control) *p* = 0.954) or gender (25/53 female and 28/53 male in LSTV and control group). In the LSTV group, *n* = 10 (18.9%) were classified as Catellvi type Ia, *n* = 6 (11.3%) as Catellvi type Ib, *n* = 14 (26.4%) as Catellvi type IIa, *n* = 9 (17.0%) as Catellvi type IIb, *n* = 5 (9.4%) as Catellvi type IIIa, *n* = 2 (3.8%) as Catellvi type IIIb, and *n* = 7 (13.2%) as Catellvi type IV.

### 3.2. Prevalence

The prevalence of LSTV was 6.5% (53/819), of six lumbar vertebrae was 1.3% (11/819), and of four lumbar vertebrae was 1.1% (9/819) in our study population.

### 3.3. Lumbosacral Transitional Vertebrae Versus Control Group

The LSTV group (*n* = 53) significantly differed from the matched control group (*n* = 53) in the characteristics PI and STA, whereas PR did not (Table 2).

### 3.4. Six Lumbar Vertebrae

In the LSTV subgroup with 6 LV (*n* = 11) (we performed the measurement of PR, PI and STA on both the superior endplates of the counted S1 (true L6) and S2 (true S1) (counted from the first non-rib-bearing vertebra, assuming L1). In the LSTV subgroup 6 LV (*n* = 11) (measurement point S2/true S1) there was a significant difference between PI and STA, but not PR, compared to its matched control group (*n* = 11) (Table 3; 6 LV S2). When comparing the subgroup 6 LV (*n* = 11) (measurement point S1/true L6) with the matched control group (*n* = 11), there was a significant difference only in the STA (*p* = 0.01), but not in PI (*p* = 0.286) and PR (*p* = 0.182) (Table 3; 6 LV S1).

We were not able to demonstrate any significant difference in the subgroups 6 LV between the two measurement points S1 (true L6) and S2 (true S1) described above with regard to PR (*p* = 0.680), PI (*p* = 0.680), and STA (*p* = 0.680). In addition, PR (*p* = 1.0), PI (*p* = 0.151), and STA (*p* = 0.254) showed no significant difference between the LSTV subgroup (6 LV S2) (*n* = 11) and the LSTV subgroup without six lumbar vertebrae (*n* = 42).

### 3.5. Four Lumbar Vertebrae

Similar results, according to the 6 LV subgroup, with a significant difference between PI (*p* = 0.021) and STA (*p* = 0.011) but not PR (*p* = 0.678), were found in the LSTV group with four lumbar vertebrae (*n* = 9) (Figure 5) and a matched group (*n* = 9) (Table 4). Also, PR (*p* = 0.448), PI (*p* = 0.771) and STA (*p* = 0.448) showed no significant difference between the LSTV subgroup 4 LV (*n* = 9) and the LSTV subgroup without four lumbar vertebrae (*n* = 44).

### 3.6. Fixed Anatomical Spinopelvic Parameter Pending the Degree of LSTV Expression

**Figure 6:**
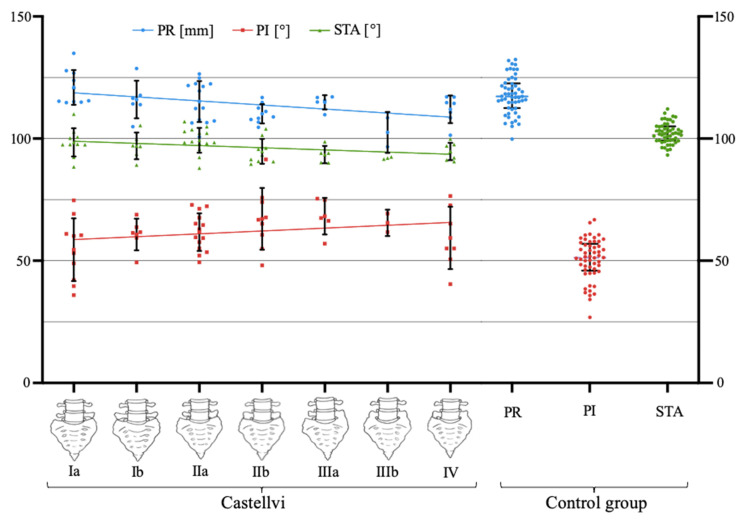
Relationships of PR (mm), PI, and STA (degree) with Catellvi classification and box-plotted values for PR (mm), PI, and STA (degree) in the control group.

Our results show significant negative correlation between the degree of LSTV expression (according to Castellvi classification Table 1) (Figure 6) and PR (*r* = −0.268; *p* = 0.008*) and STA (*r* = −0.232; *p* = 0.022*), as well as a significant positive correlation with PI (*r* = 0.201; *p* = 0.047*) (* indicates significance).

### 3.7. Accuracy of the Radiographic Measurement

The interrater reliability was assessed with 0.92 in Spearman’s rank correlation coefficient, and is therefore considered excellent.

## 4. Discussion

It is well recognized that spinal sagittal balance and functional spinopelvic parameter are decisive elements in describing spinal alignment [13]. Consideration of the sagittal spinal profile and pelvic morphology are key factors in spinal surgery, with the aim of restoring the sagittal alignment [13,14,20,21,29]. Restoration of the sagittal profile is directly related to the improvement of pain and function after spine surgery for various disease states [30,31]. However, it has been discussed that the spinal profile, and thus the functional spinopelvic parameters, are highly variable, and changes both in the short term during daily activities and in the long term due to degeneration are possible [22]. In particular, constant anatomical parameters of the pelvis have been focused on mitigating the influence of functional changes in spinopelvic parameters. In this respect, it has already been shown that there is a close correlation between the pelvic shape, represented by the fixed spinopelvic parameters (PR, PI, STA), and the lumbar degeneration types [25]. PI combined with PR and STA determines anatomical and non-posture-related spinopelvic orientation [17,20]. The posture-dependent pelvic parameters sacral slope and pelvic tilt are related to PI, as well as the degree of lumbar lordosis correlates with PI [14,32]. The PI-LL mismatch influences the selection of the surgical procedure, as well as its invasiveness. Ponte corrective osteotomy, Smith–Peterson osteotomy, pedicle subtraction osteotomy, and vertebral column resection are all to restore sagittal balance; their planning is therefore also influenced by spinopelvic parameters [31,33].

We showed in this distinct population of LSTV patients that PI increased significantly in the LSTV, 6 LV, and 4 LV groups, while STA decreased significantly and PR showed no significant differences. In addition, in the 6 LV patients a significant difference in the PI compared to the control group was demonstrated only at the measurement point S2 (true S1) and not S1 (true L6). There have been no reports studying the relationship between LSTV and the fixed anatomical spinopelvic parameters. Furthermore, there are currently no normative values for lumbarization and sacralization subjects with regard to fixed anatomical spinopelvic parameters in the literature, in contrast to patients without transitional disorders [34]. Therefore, LSTV should be safely detected in patients with alterations of the sagittal profile prior to surgical planning, and its strong impact on spinopelvic alignment, especially the significant increased PI, should be taken into account.

We have determined the prevalence of LSTV, lumbarization, and sacralization in our collective of 819 patients who received an abdomen–pelvis CT scan. The prevalence of LSTV was 6.5% (53/819), of 6 LV was 1.3% (11/819), and of 4 LV was 1.1% (9/819) in our central European study population. Whereas Dzupa et al. observed a distinctly higher LSTV prevalence of 27.6% (417/1513) in a central European study population using pelvic X-ray [9], Tang et al. have found a 15.8% (928/5860) prevalence of LSTV in a Chinese Han population by X-ray of the lumbarvertebral column. This might be due to differences in prevalence in the Chinese population, or to the underlying evaluation of lumbar radiographs rather than CT scans [2]. The investigation by Sekharappa et al. in an Indian study population revealed a similar prevalence (8.1%; 81/1000) of LSTV in urological patients as in our cohort of patients (6.5%). In contrast, the LSTV prevalence of 14% (140/1000) in the same study was significantly higher in patients visiting the spine outpatient clinic [12]. This leads to the assumption that there may be a selection bias in the prevalence of LSTV. The studies by Yokoyama (Japanese study population) and Price (Japanese and French study population) et al. reported a higher prevalence of 6 LV, at 17.4% (29/167) and 4.1% (11/268), respectively, compared to our prevalence of 1.3% [8,27]. The differences may be due to the examination of the Japanese population (Yokoyama and partly Price) and the small study population (*n* = 167 for Yokoyama, and *n* = 268 for Price). In contrast, Abola et al. identified a considerably lower prevalence of 6 LV (0.8%; 23/969) and 4 LV (1.8%; 54/969) in their cadaver study (white and African-American study population), comparable to our results (1.3% for 6 LV and 1.1% for 4 LV) [26].

There was a significant increase in PI and decrease of STA in the LSTV group (*n* = 53) compared to the matched control group, without a significant difference in PR. Accordingly, these findings were made in our subgroups with 6 LV (*n* = 11) and 4 LV (*n* = 9). This is consistent to the investigations by Price and Yokoyama et al., which showed increases in PI in patients with 6 LV [8,27]. In contrast, Abola et al. could not find a significant difference regarding PI in patients with 6 LV, and contradicting our results reporting a significant decrease of PI in patients with 4 LV [26]. Interestingly there were no significant differences regarding PR, PI, and STA when comparing the 6 LV (*n* = 11) or the 4 LV subgroup (*n* = 9) with the remaining LSTV subgroups (*n* = 42 and *n* = 44, respectively). This underlines the relevance of the reliable identification of LSTV for the correct assessment of spinopelvic parameters.

Comparing PR, PI, and STA in the 6 LV subgroup at the measurement points S1 and S2 with the matched control group, a significant difference was detected in PI (S2) and STA (S2), but also in STA (S1). However, we were not able to demonstrate any significant differences in the subgroup with 6 LV between the two measurement points (S1/S2), counted from the first non-rib-bearing vertebra to the superior endplate of S1 (true L6) and S2 (true S1) with regard to PR, PI, and STA. According to Price et al. who assessed the pelvic incidence of 11 patients with 6 LV at the measurement points S1 (true L6) and S2 (true S1), and as in our study (6 LV *n* = 11), significant differences were recorded only for the measurement point S2 for PI compared to a control group [27]. In contrast, Kyrölä et al. (Finnish study population: 6 LV = 7.7%; 60/775) found a significant difference between PI and the control, S1(true L6), and S2 (true S1) groups, in a collective of patients with 6 LV (*n* = 60) [28]. This underlines the importance of the accurate study of spinopelvic parameters in the LSTV cohort, and the impact of the measurement point in patients with an abnormal number of lumbar vertebrae. The measurement of PI-LL mismatch for evaluating and restoring the sagittal profile is significantly affected by choosing the measurement point in patients with six lumbar vertebrae.

Although Khalsa et al. revealed high variability between spine surgeons in the measurement of spinopelvic parameters, we demonstrated a very good interrater reliability of 0.92 for the measuring orthopedic surgeons [35].

Some limitations need to be mentioned. There is a risk of a falsely assumed LSTV or additional vertebrae (“pseudo-lumbarization”) due to the vertebral column not being fully imaged, and counting from C2 not possible. The risk of incorrectly overestimating the prevalence of 6 LV is considered small in our study, as our prevalence is lower than other studies. Mistakenly assuming LSTV might be balanced out by the use of CT imaging as the gold standard for the diagnosis of LSTV [36]. The first vertebra without a rib was defined as L1, but lumbar rips occurring with a prevalence of 1% may cause bias, so it has to be noted as a limitation [37]. An evaluation of spinopelvic functional or global spinal balance parameters as lumbar lordosis, sacral slope, pelvic tilt, or sagittal vertical axis was not performed, because we investigated supine abdomen–pelvis CT. The statistical analysis may contain biases due to the small number of samples in the 4 LV and 6 LV groups.

The aim of our study was to define, for the first time, normative values of the fixed anatomical spinopelvic parameters (PR, PI, and STA) in a patient cohort with LSTV. PI was significantly increased in the LSTV group, and in the subgroups with 6 LV and 4 LV. STA was significantly decreased in the LSTV group, and in the subgroups with 6 LV and 4 LV. PR showed no significant differences in the LSTV group or in the subgroups with 6 LV and 4 LV, compared to the matched control group. In patients with 6 LV, the PI measurement showed only significant differences to the control group at measurement point S2 (true S1), and not at S1 (true L6), compared to the control group. Accordingly, the reliable identification of LSTV, as well as the proper selection of measurement points is of great clinical relevance, due to its significant influence on pelvic morphology and spinopelvic parameters. The considerable value of PI and LL in the preoperative planning of the restoration of spinal sagittal balance should lead to increased attention when measuring the spinopelvic parameters in LSTV patients.

## Figures and Tables

**Table 1:** Radiographic lumbosacral transitional vertebrae (LSTV) classification according to Castellvi [4].

Castellvi Type	Definition
Type I: dysplastic transverse process	Unilateral (A) or bilateral (B) dysplastic transverse process with a height >19 mm
Type II: incomplete lumbarization/sacralization	Enlarged transverse process with unilateral (A) or bilateral (B) pseudoarthrosis with the adjacent sacral ala
Type III: complete lumbarization/sacralization	Enlarged transverse process, which has a unilateral (A) or bilateral (B) complete fusion with the adjacent sacral ala
Type IV: mixed	Type II on one side and type III on the contralateral side

**Table 2:** Comparison between the LSTV (*n* = 53) and the matched control group (*n* = 53) in terms of PI (pelvic incidence), PR (pelvic radius), and STA (sacral table angle) (SD: standard deviation). For the 6 LV patients, we have considered the measurement point S2 (true S1) in the calculation. Significance level was assessed at *p* < 0.05, with * indicating a significant difference.

	n	Mean	SD	Range	*p*-Value
PR LSTV	53	114.5	7.6	96.6–134.9	0.051
PR Control	53	117.8	7.6	99.8–132.4	
PI LSTV	53	61.6	10.8	35.8–91.5	0.001 *
PI Control	53	50.5	8.4	26.8–66.8	
STA LSTV	53	96.7	5.2	87.9–110.0	0.001 *
STA Control	53	102.2	4.2	93.3–121.1	

**Table 3:** Comparison between the LSTV subgroup with 6 lumbar vertebrae (*n* = 11) measured at S1 (6 LV S1) and S2 (6 LV S2) and the matched control group (*n* = 11), in terms of PR (pelvic radius), PI (pelvic incidence), and STA (sacral table angle) (SD = standard deviation). *p*-values are defined between the matched control group and the respective measurement point, with * indicating a significant difference. The significance level was assessed at *p* < 0.05.

	n	Mean	SD	Range	*p*-Value
PR 6 LV S1	11	120.0	11.2	107.8–138.6	0.182
PR 6 LV S2	11	114.2	4.5	107.8–122.5	0.859
PR Control	11	113.7	4.7	107.2–121.5	
PI 6 LV S1	11	54.6	14.1	35.0–75.4	0.286
PI 6 LV S2	11	65.3	8.5	48.1–73.4	0.010 *
PI Control	11	48.5	10.2	26.8–57.9	
STA 6 LV S1	11	97.2	4.2	89.2–109.4	0.010 *
STA 6 LV S2	11	95.1	4.2	89.2–101.4	0.004 *
STA Control	11	103.0	4.0	99.1–110.1	

**Table 4:** Comparison between the LSTV subgroup with four lumbar vertebrae (*n* = 9) and the matched control group (*n* = 9) in terms of PR (pelvic radius), PI (pelvic incidence), and STA (sacral table angle) (SD = standard deviation); * indicates a significant difference. The significance level was assessed at *p* < 0.05.

	n	Mean	SD	Range	*p*-Value
PR 4 LV	9	111.3	7.6	96.6–117.1	0.678
PR Control	9	107.3	4.8	113.1–121.6	
PI 4 LV	9	62.5	8.8	50.6–75.4	0.021 *
PI Control	9	52.8	6.5	38.5–57.9	
STA 4 LV	9	95.2	3.2	91.2–99.8	0.011 *
STA Control	9	102.8	4.0	99.1–109.2	

## Data Availability

The data presented in this study are available on request from the corresponding author. The data are not publicly available due to patient data protection reasons in line with the ethics vote.
